# Moving towards universal health coverage for mental disorders in Ethiopia

**DOI:** 10.1186/s13033-019-0268-9

**Published:** 2019-02-25

**Authors:** Charlotte Hanlon, Atalay Alem, Crick Lund, Damen Hailemariam, Esubalew Assefa, Tedla W. Giorgis, Dan Chisholm

**Affiliations:** 1 Centre for Global Mental Health, Health Service and Population Research Department, Institute of Psychiatry, Psychology and Neuroscience, King’s College London, London, UK; 2 Department of Psychiatry, School of Medicine, College of Health Sciences, Addis Ababa University, Addis Ababa, Ethiopia; 3 Alan J Flisher Centre for Public Mental Health, Department of Psychiatry and Mental Health, University of Cape Town, Cape Town, South Africa; 4 School of Public Health, College of Health Sciences, Addis Ababa University, Addis Ababa, Ethiopia; 5 Department of Economics, Faculty of Arts and Social Sciences, The Open University, Milton Keynes, UK; 6 Office of the Minister, Federal Ministry of Health, Addis Ababa, Ethiopia; 7 Department of Mental Health and Substance Abuse, World Health Organization, Geneva, Switzerland

**Keywords:** Universal health coverage, Health financing, Mental health, Mental disorders, Financial coverage, Health insurance, Ethiopia, Health expenditure, Sub-Saharan Africa

## Abstract

**Background:**

People with mental disorders in low-income countries are at risk of being left behind during efforts to expand universal health coverage.

**Aims:**

To propose context-relevant strategies for moving towards universal health coverage for people with mental disorders in Ethiopia.

**Methods:**

We conducted a situational analysis to inform a SWOT analysis of coverage of mental health services and financial risk protection, health system characteristics and the macroeconomic and fiscal environment. In-depth interviews were conducted with five national experts on health financing and equity and analysed using a thematic approach. Findings from the situation analysis and qualitative study were used to develop recommended strategies for adequate, fair and sustainable financing of mental health care in Ethiopia.

**Results:**

Opportunities for improved financing of mental health care identified from the situation analysis included: a significant mental health burden with evidence from strong local epidemiological data; political commitment to address that burden; a health system with mechanisms for integrating mental health into primary care; and a favourable macro-fiscal environment for investment in human capabilities. Balanced against this were constraints of low current general government health expenditure, low numbers of mental health specialists, weak capacity to plan and implement mental health programmes and low population demand for mental health care. All key informants referred to the under-investment in mental health care in Ethiopia. Respondents emphasised opportunities afforded by positive rates of economic growth in the country and the expansion of community-based health insurance, as well as the need to ensure full implementation of existing task-sharing programmes for mental health care, integrate mental health into other priority programmes and strengthen advocacy to ensure mental health is given due attention.

**Conclusion:**

Expansion of public health insurance, leveraging resources from high-priority SDG-related programmes and implementing existing plans to support task-shared mental health care are key steps towards universal health coverage for mental disorders in Ethiopia. However, external donors also need to deliver on commitments to include mental health within development funding. Future researchers and planners can apply this approach to other countries of sub-Saharan Africa and identify common strategies for sustainable and equitable financing of mental health care.

**Electronic supplementary material:**

The online version of this article (10.1186/s13033-019-0268-9) contains supplementary material, which is available to authorized users.

## Background

The drive for universal health coverage (UHC), articulated as a key target in the sustainable development goal for good health and wellbeing, is motivated by the desire for health equity across the globe [1]. To achieve UHC, there is a need for both population coverage of adequate quality services and financial coverage or risk protection. People with mental disorders who live in low- and middle-income countries (LMICs) have low access to quality mental health care and are consequently vulnerable to suffering and disability [2], human rights abuses [3], stigma and discrimination [4], impoverishment [5] and premature mortality [6]. The neglect of mental health care globally [7], combined with catastrophic healthcare costs due to high out-of-pocket expenditure, the economic costs of being unable to work, household costs of caring for someone with mental health problems and the limited economic opportunities due to social marginalisation [8], means that people with mental health problems and their families are at great risk of being ‘left behind’ by development initiatives such as UHC [9].

In Ethiopia, service coverage and financial protection for people with mental disorders is limited, while the adverse economic consequences of these disorders on households are pronounced. People with severe mental disorders (including schizophrenia and bipolar disorder) are more likely to be unemployed [10] and their households are at elevated risk of severe food insecurity compared to the general population [11]. The economic burden on households with a person with bipolar disorder was found to be higher than for households with a person with a chronic physical disorder (diabetes, asthma or hypertension) [12]. Caregivers report that the economic burden of mental disorder is their main concern [13], which is alleviated when care is made available and symptoms resolve [14]. However, even when mental health care is made geographically accessible by integration into primary care, the costs of conveying a family member who has acute mental disturbance and the need for ongoing payment for psychotropic medication force people to drop out of care [15]. Inadequate financial coverage is thus a major barrier to accessing mental health care in Ethiopia.

The Emerging mental health systems in low and middle-income countries (Emerald) project aimed to identify key health system barriers to, and solutions for, the scaled-up delivery of integrated primary mental health care in six LMICs (Ethiopia, India, Nepal, Nigeria, South Africa and Uganda), and by doing so to improve mental health outcomes in a fair and efficient way [16]. In this paper we focus on the core health system inputs and funding mechanisms needed to improve mental health coverage and meet the mental health needs of the Ethiopian population. The objectives of this paper are to set out:

1. An organising framework for planning more equitable and sustainable mental health financing in Ethiopia;
2. A situational analysis of where Ethiopia lies, both with respect to the key dimensions of UHC but also broader health system characteristics and the macroeconomic and fiscal environment;
3. Main findings from in-depth interviews with national experts on health financing, equity and potential strategies for increased financial protection for people with mental disorders;
4. Proposed strategies for moving towards UHC for people with mental disorders in Ethiopia, building on the findings of (2) and (3).

## Methods

### Analytical framework

The Emerald project has undertaken a range of research activities along the pathway to determining strategic financing needs for the future scale-up of mental health care, including estimation of the costs and impacts of scaled up mental health care using the newly-developed OneHealth tool [17] and assessing the economic burden of mental disorders on households, in terms of catastrophic healthcare expenditure and impoverishment using a multi-country survey [16]. Building on this work, to ascertain options for adequate, fair and sustainable mental health financing in Ethiopia, a framework was developed by the project partners, informed by existing health system and sustainable financing frameworks [18, 19], as described in Table 1.

**Table 1:** Dimensions of a framework to identify options for fair, adequate and sustainable financing of mental health care in Ethiopia

Dimensions of assessing financing options	Explanation of approach
Projected public health and economic consequences of mental health problems	Synthesis of the evidence base from Ethiopia on epidemiology and impact of mental health problems, with consideration of future trends based on demographic and epidemiological transitions
Current and proposed governance, service delivery and financial protection arrangements for the treatment and prevention of mental health problems	The status of health system leadership and management, accountability, service configurations and human resourcing, and financial protection for health in general, and mental health in particular
The current and projected macro-fiscal situation	The past and present economic status of the country, including economic growth, unemployment, debt, fragility, health expenditure
Projected resource needs for mental health problems	Estimation of the human, technical and financial resources required over time to scale-up services and move towards universal health coverage for people with mental disorders (the OneHealth tool was used)
Identification and selection of appropriate financing mechanisms	Identification and assessment of potential mechanisms for moving towards more equitable and sustainable mental health financing in the Ethiopian context

### Data sources

The data sources to inform the dimensions of the framework included (1) a situational analysis of publicly available documents, and (2) in-depth, structured discussions with expert stakeholders.

#### Situational analysis

The situation analysis was initially conducted in July 2015 by research assistants working with the Ethiopia team and co-author (DC) and updated in June 2018 by co-author EA. The template used for the situation analysis is included in Additional file 1. The situation analysis template included collection of publicly available data to map onto the framework dimensions, as follows: (A) burden of disease (country-specific estimates for mental health and substance use-related mortality, disability-adjusted life-years and years lived with disability; economic burden); (B) mental health system, in terms of governance (mental health legislation, policy, plans and programmes), financing (expenditure on aspects of mental health care), human resources (mental health specialists and potential task-sharing workforce in general health care), availability of services (mental health treatment gap, specialist in-patient and out-patient care, extent of scale-up of task-shared care); (C) health system in general, in terms of key health indicators, financing (total, public and private), delivery and access, service coverage; (D) macro-economic situation (size of the economy, growth, government finances, debt/borrowing; (E) level of development, in terms of poverty and inequality, development indicators and social protection and labour; (F) political situation, in terms of polity, stability and control of corruption, and (G) demographic situation (current and projected).

A literature review was undertaken to identify relevant Ethiopia-specific data on health financing, the economic burden of mental disorders and efforts to expand mental health care coverage. See Additional file 2 for the detailed search strategy, conducted in PubMed and Medline with no date restriction (date of search 21st June 2018). A total of 191 records was returned by the search. Following title and abstract review by EA and CH, 15 full texts of papers were obtained, of which four were considered to provide relevant data in relation to the framework dimensions and were included in the analysis. See Additional file 3 for flow diagram of paper selection.

The grey literature was searched by (1) purposively identifying relevant data sources from government institutions and non-governmental organisations working in the area of health financing and health care in Ethiopia, as well as Ethiopia-specific data produced by global actors, including the World Bank and United Nations organisations (World Health Organization, United Nations Development Programme, International Labour Organisation), and (2) searching Google for reports about Ethiopia in relation to the framework dimensions. A total of 28 reports and four databases were identified as being relevant to the framework dimensions and were included in the analysis.

Findings from the situational analysis were used to develop a SWOT analysis of the situation in Ethiopia, summarised in tabular form into threats and opportunities, and as a narrative synthesis relating to the framework dimensions. Based on the information collected under each assessed dimension, the team categorised prospects for scaling up investment in mental health into: ‘poor’, ‘moderate’ or ‘good’.

#### Stakeholder views

In-depth interviews were carried out with key informants from the Ministries of Health and Finance, non-health/non-finance state actors and non-state stakeholders, using a Mental Health Financing Diagnostic Tool developed by the Emerald project consortium which covered the framework dimensions outlined above (see Additional file 3 for the topic guide). The interviews were conducted from January to May 2015. Five senior health and financing policy makers were interviewed, including a former Minister of Health, a senior mental health advisor within the Ministry of Health, a senior academic health economist and representatives from the Ministry of Health resource mobilisation department and the national health insurance agency. The key informants were identified initially through existing contacts with the Ministry of Health and then using a snowballing approach. The initial plan was to interview 8–10 key informants or continue recruitment until saturation was attained; however, given the low profile of mental health care within Ethiopia, it was difficult to identify informants with the required expertise. Nonetheless, given the experience and expertise of the key informants identified, saturation on key themes was achieved.

The main findings of the situational analysis were presented to the key informants and used to probe for potential financing strategies suitable for the Ethiopian context. Interviews were conducted by CH and AA in English in a location convenient to the interviewee, usually a private office at their place of work. All interviews were audio-recorded, with the permission of the participants, and transcribed and analysed in English. The duration of interviews ranged from 36 to 53 min. A framework analysis approach was used [20], with pre-specified high level themes of: (1) perceived challenges and constraints to increasing public health financing, (2) options for change, and (3) key elements/criteria for improved public health financing. The data were analysed thematically under these over-arching themes using an Excel spreadsheet. All analysis of the qualitative data was conducted by CH.

#### Identification of potential strategies

Potential strategies for adequate, fair and sustainable financing of mental health care in Ethiopia were then developed, based on the constraints and opportunities of the Ethiopian setting identified through the situation analysis and the in-depth interviews. In broad terms, there are three key health system financing functions: revenue generation; pooling of funds; and purchasing. Consideration was first given to the three options that governments have for raising revenue for the health sector: bilateral and multilateral financing, domestic financing, and innovative financing [21]. Innovative sources to fund health include taxes and levies, voluntary contributions and market-based financial mechanisms [21]. Within these overall financing options, potentially appropriate financing strategies were identified on the basis of a range of factors, including the potential for raising revenue, increasing equity and social protection, and offering stable and sustainable funding flows [22]. These strategies, together with the underpinning rationale, were discussed within the Ethiopia Emerald team and email exchanges allowed for input from Emerald consortium health systems experts. At the October 2017 meeting of the Emerald consortium, the integrated findings from different aspects of the Emerald financing work package were presented which allowed triangulation of the contextual validity of the proposed strategies.

## Results

### Situational analysis

#### Mental health burden

In Ethiopia’s population of around 100 million, neuropsychiatric disorders are estimated to account for 5.8% of the disease burden [23]. In 2016, depression alone accounted for 6.2 percent of total years lived with disabilities (YLD) [24], ranking fourth out of all causes of YLD in Ethiopia [25]. Although there are no data on trends in the prevalence of mental health problems over time, the burden of depression in Ethiopia is estimated to have increased by 39.6% from 2005 to 2016 due to demographic transition [25]. In a predominantly rural area, mental disorders were estimated to be responsible for 11% of the total disease burden, with schizophrenia and depression among the top ten most burdensome conditions [26]. Epidemiological studies have produced Ethiopia-specific estimates of the prevalence of priority mental disorders: schizophrenia (lifetime) 0.5% [27], bipolar disorder (lifetime) 0.5% [28], alcohol dependence (12 month) 1.5% [29], depression (12 month) 5.0% and childhood mental illnesses (12 month) 12–25% [30]. These prevalence estimates are in keeping with those seen in population studies from other African countries [2, 31].

An analysis of cost-effectiveness of treatment for mental disorders in Ethiopia indicated that treatments for depression had mid-range cost-effectiveness compared to other interventions [US$ 457–1026 per disability-adjusted-life-year (DALY) averted] and treatments for schizophrenia and bipolar disorders were less cost-effective (US$ 1168–3739 per DALY averted) [32].

#### Health system

Ethiopia has a three-tiered health care delivery system. The Primary Health Care Unit is comprised of a primary hospital (1 per 60–100,000 population), health centres (1 per 15–25,000), and satellite health posts (1 per 3–5000) connected by referral. Level two is a general hospital covering 1–1.5 million people, and level three is a specialized hospital covering 3.5–5 million.

The most recent estimates of the healthcare workforce indicate 0.044 physicians, 0.097 health officers and 0.84 nurses [33] per 1000 people, which is lower than other countries in East Africa [34]. However, Ethiopia has increased its coverage of community health workers (to 0.423 per 1000 people) because of the Health Extension Program launched by the Federal Ministry of Health (FMoH) in 2004. As of 2015, the program had trained and deployed over 42,000 paid female health workers, with a ratio of 1 health extension worker (HEW) per 2500 population [35]. The community health extension worker upgrading programme includes a package on mental health care which focuses on early detection, prevention and promotion [36]. A network of community health volunteers, referred to as the Health Development Army (HDA), has also been established to support the Health Extension Program with dissemination of health information and promotion of uptake of health care [37]. The HDA is estimated to involve approximately three million women.

With improved access to health services, Ethiopia was able to achieve the Millennium Development Goals on child mortality and sustainable access to safe drinking water [38]. However, maternal care indicators (mortality, antenatal coverage and births attended by skilled health staff) lag behind SDG targets [1, 39] and are poorer than other countries in the region [39].

Inequity in access to health care is evident. Recent figures show that, while 85.8% of the richest quintile obtain antenatal care from a skilled provider, only 49.8% of the poorest quintile obtain the same service. Similarly, percentage of live births delivered by a skilled provider was 66.9% and 13.1% among the richest and poorest quintile, respectively [39].

#### Governance and leadership

In recent years, FMoH has demonstrated some political will to addressing the significant mental health burden in the form of policies, plans and programmes to promote mental health of the population. In 2012, FMoH launched the national mental health strategy (NMHS), which aimed to develop mental health services that are “decentralized and integrated at the primary health care level” [30]. The NMHS is currently under revision. At present, there is no legislation to protect the rights of people with mental health problems in Ethiopia [40]. Other programmes are also underway in support of mental health. From 2011 to 2014, Ethiopia was one of the six pilot sites of the WHO Mental Health Gap Action Programme (mhGAP) [41]. Subsequently, the FMoH launched a plan to scale up mental health care integration into primary care based on the mhGAP model. The emphasis on task-shared care has been accompanied by expansion of training programmes for specialist mental health workers, including psychiatric nurses, Master’s level psychiatric practitioners, clinical psychologists and psychiatrists. However, the numbers of specialists fall short of recommended minimum levels [42]. Furthermore, current approaches to training may not equip specialists adequately for leadership roles, service planning, and training and supervising delivery of mental health care by general health care workers [42]. The expansion of mental health care is also hampered by the absence of a national level organisation to represent current and potential mental health service users. There are low levels of service user involvement in planning, developing and monitoring services [42].

Broader health sector policies and plans are supportive of both scaling up evidence-based mental health services and sustainable financing. Ethiopia’s national health policy (NHP) defines a series of priorities, among which is the development of curative and rehabilitative components of health, including mental health [43]. The NHP was initially implemented through a series of consecutive 5-year Health Sector Development Plans (HSDP). In HSDP-IV, Ethiopia developed a national health insurance strategy. The strategy involves social health insurance (SHI) to cover employees in the formal sector and community-based health insurance (CBHI) to cover the rural population and urban informal sector for a range of common health conditions. From 2010, the focus has shifted to 5-year Health Sector Transformation Plans (HSTP). In HSTP-1, one of the performance measures related to ‘improving equitable access to quality health services’ was linked to the scale-up of mental health care, with a target of ‘making mental health services available in every district in Ethiopia by the end of 2020’ [37].

Piloting for CBHI began in 2011 and scale up of the scheme commenced in 2014 [44]. By 2016/17, 377 districts had been nominated for implementation, out of which 248 districts had begun enrolling participants and providing services [45]. So far, from the total number of eligible households in the districts that have implemented the scheme, on average 36 percent are enrolled [45]. From those enrolled, approximately 79 percent were paying members while 21 percent were non-paying members from the poorest sector of society [45]. A small number of studies have been published on the CBHI pilot. One study finds a high willingness to participate in the scheme [46]. Another shows an increase in general outpatient care services but no significant effect on general inpatient care [47], which would support scale up of community-based mental health care. Annual renewal of participation has been found to be high, with increasing registration of households over time and generally high levels of satisfaction [48]. The Social Health Insurance scheme has not yet been implemented, with the focus currently on preparation and capacity building to roll out the scheme [45]. However, using discrete choice experimental methodology, SHI preferences of government employees were for lower premiums (1.52%) than those proposed (3.0%) and more comprehensive coverage including public and private providers [49]. Other studies have found higher willingness to pay but may have been more susceptible to social desirability bias [50].

#### Macro-fiscal environment

##### Macroeconomic environment

The GDP per capita of the Ethiopian economy was US$ 706.8 in 2016, which was less than half that of the sub-Saharan African average of US$ 1467.3 [51]. However, with 7.5 percent average annual growth of GDP per capita over 2009–2015, Ethiopia is among the five fastest growing economies in the world [52]. Tight monetary policies and a slowdown in global commodity prices contained annual inflation at 9.5% in 2016, a significant decline from 33.5% in 2012 [51]. The International Labour Organization (ILO) estimates that 81.2% of the working-age population is engaged in the labour market [53]. The unemployment rate is currently 5.3%, with estimates from 2013 indicating that over half suffer from long-term unemployment [53]. In 2017, within total employment, 88% were in vulnerable employment, meaning they are unpaid family workers or own-account workers [51]. In addition to being an important indicator of macroeconomic strength, unemployment and poor quality employment are risk factors for mental health problems [54].

As measured by Ethiopia’s national poverty line (US$ 0.60 a day), the incidence of poverty has declined from 44.2% in 2000 to 29.6% in 2010 [55]. Using the international poverty line of US$ 1.90 per day per capita, the share of the population below the poverty line was 26.7% in 2015 [55]. However, with rapid population growth, the absolute number of people below the line is unchanged in the past 15 years, at 25 million [56]. Using a multidimensional poverty headcount, even more Ethiopians are considered poor, with 88.2% suffering deprivations in at least one-third of the weighted indicators across health, education and standard of living, with an additional 6.7% living near multidimensional poverty [57].

##### Fiscal context

Both general government revenue and expenditure as a proportion of GDP are relatively low in Ethiopia, with government revenue of 15.2% of GDP in 2015/16 [58]. The tax revenue as the percentage of GDP was 12.5% [58]. Deficit in Ethiopia is relatively low, estimated at 2.4% of GDP in 2015/16, reflecting conservative government spending [58]. Especially important to financial sustainability is the level of general government gross debt, which was 55.4% of GDP in 2015/16 and is projected to slightly increase in the coming few years. As a result of export underperformance, declining reserves and approaching maturity of past debt obligation, the International Monetary Fund opined the risk of debt distress is high [58].

##### Government priority setting for health

Total health expenditure (THE) per capita was US$ 28.65 in 2013/14 [59], which is just over a quarter of the regional average of US$ 84.9 [51]. This is below Ethiopia’s HSDP-IV per capita base-case spending target of US$ 32 [60], and below global expert panel recommendations, like that of the Taskforce on Innovative International Financing for Health recommendation of US$ 44 per capita by 2015 for low income countries [61]. As a proportion of GDP, total health expenditure was 4.1% in 2015, which is also below the regional expenditure of 5.4% of GDP [51].

General government health expenditure (GGHE) as a proportion of general government expenditure (GGE) was 6.65% in 2013/14 [59], which is below the agreed level of commitment towards the health sector (15% of total budget) articulated in the Abuja Declaration [62]. As a proportion of GDP, general government health expenditure (GGHE) has hovered around 1.4% for the past 10 years [63], which is well below international recommendations of at least 5% [64]. Furthermore, GGHE accounted for a little over quarter of total health expenditure (THE) (approximately 30%) [59]. External resources make up around 36% of THE, with the remaining 34% from private health expenditure including household out-of-pocket spending, private sector employers, private insurance schemes and others [59]. In the absence of well-developed alternative financing mechanisms, the proportionally high percentage of private health expenditure indicates high financial risks for individuals and barriers to accessing health services. This is reflected by the high percentage of THE (33%) accounted for by out-of-pocket payments [59]. This level is higher than what is expected to ensure financial protection (which is 20%) [65] and the government has set a target to reduce out-of-pocket health expenditures to less than 15% by 2020 [37].

The government is the main source of funding for the care of people with severe mental health disorders in Ethiopia, but data on mental health expenditure are not collected [40]. For African countries and low-income countries, respectively, the median percentage of health budget allocated to mental health is 0.62% and 0.53%, which can be used as a proxy for Ethiopia [23].

Based on the preceding situational analysis across the four assessed domains, certain opportunities for, and threats to, the scaleup of investment in mental health become apparent. These are summarised in Table 2. On the basis of the SWOT summary analysis, it is concluded that important opportunities do exist in Ethiopia because of:

**Table 2:** Opportunities for, and threats to, scaling up investment in mental health in Ethiopia

Domain	Opportunities for mental health service scale up	Threats to mental health service scale up	Overall prospects
Mental health burden			
Public health burden	High public health burden of mental disorders, well-documented with Ethiopia evidence Increasing burden due to demographic transition	Weak information systems which do not allow quantification of disorder-specific healthcare utilisation Population demand for mental health care is currently low	Good
Health system			
Service availability and access	Three-tiered system with strong primary care units Recent investments in facilities and workforce, including specialist mental health workers High coverage of health extension workers Gains in child mortality Integration of mental health care into the new Ethiopia primary health care guide (PHCG) Mental health and NCDs integrated within the health extension worker upgrading training and expanded set of core service packages for level IV HEWs	Poor access to basic care (i.e. antenatal care) and high maternal mortality rate Inequity in access between rich/poor and urban/rural Specialist mental health personnel are concentrated in urban areas Limited supervision of task-shared care by mental health specialists Mental health care checklists and indicators not integrated into HEW reporting Low mobilisation and involvement of current and potential mental health service users in planning and developing services	Moderate
Governance and leadership			
Political will	Mental health on the political agenda in FMoH Regional Health Bureaus committed to new Ethiopia PHCG which includes mental health	Limited Regional Health Bureau buy-in and capacity for mental health care expansion	Good
Mental health policies and plans	National mental health strategy with plans for integrated care 12-year plan with specific budgets and targets PRIME demonstration site providing a model for successful implementation Proposal for multi-sectoral National Institute of Mental Health	Limited evaluation of policy implementation No legislation protecting the rights of the mentally ill National mental health strategy expired in 2016 and remains under revision Absence of a national mental health service user organisation	Moderate
Health sector plans	Mental health integrated into health sector transformation plan Development of health insurance strategy which includes priority MNS disorders; pilots show promising results New Ministry initiative for scaling up Ethiopia Primary Health Care Guidelines, which has mental health horizontally integrated	Implementation of insurance schemes behind schedule Limited follow-through on mental health targets of the health sector transformation plan (HSTP)	Good
Macro-fiscal environment			
Macroeconomic conditions	High annual GDP growth Contained inflation	High vulnerable employment High poverty headcount	Good
Fiscal context	Debt and deficit relatively low	Revenue and expenditure relatively low	Moderate
Priority setting	Government main source of total health and mental health expenditure Health a high budgetary priority	One-third of health budget from external sources THE per capita very low High out-of-pocket expenditure	Moderate

- a significant mental health burden with evidence from strong local epidemiological data;
- a political commitment to addressing that burden;
- a health system with mechanisms for integrating mental health into primary care; and
- a favourable macro-fiscal environment for investment in human capabilities.

### Stakeholder views

Findings from the in-depth interviews with stakeholders, categorised under three main themes, are summarised in Table 3. Many of the insights resonate with what was ascertained through the situational analysis, for example the opportunities afforded by positive rates of economic growth in the country and the expansion of community-based health insurance, as well as the need to integrate mental health into other priority programmes and strengthen advocacy to ensure mental health is given due attention.

**Table 3:** Overview of findings from the in-depth interviews with stakeholders

Perceived challenges and constraints to increasing public health financing	
Priority given to mental health	Inadequate financing relative to burden Limited buy-in from Regional Health Bureaus Lacking a unified advocacy effort and clear message Low population awareness and low demand
Mental health strategies and plans	Mental health is integrated within key policy documents, but implementation is inadequate Need for health extension worker checklists for reporting mental health
Financing policies and strategies	High out-of-pocket expenditure Delayed implementation of social health insurance Community-based health insurance coverage is still low Plan for ‘sin tax’ but revenue not earmarked for health External donors show little interest in mental health
Barriers to budget allocation process	Budget allocation is not driven by global burden estimates, which disadvantages mental health
Impact of macro-economic issues	Good economic growth and stable debt may increase fiscal envelope for health care, but external donors are cutting back support proportionately
Options for change for increased financing for public health	
Strengthening mental health systems	Continue to expand mental health care to address unmet need Re-invigorate task-sharing model of integrating mental health into primary care as an efficient strategy to expand access Expand efforts to counter stigma and raise awareness
Improving public health financing policies	Focus on implementation of existing policies
Financing mechanisms	CBHI and SHI for equitable increase in financial protection Commitment to community-based health insurance may expand healthcare spending overall, and mental health as part of that
Key elements/criteria for improved public health financing	
Budget planning and allocation for general and mental health	Need for sustained advocacy to improve fairness of budget allocation Better oversight of implementation to ensure that the allocated budget is spent
Engagement of participants in mental health financing	Advocacy from a broad base of stakeholders is needed
Monitoring and evaluation of health systems/financing	HMIS indicators for mental health need to be linked to financing HMIS system not adequately capturing NGO and private sector

#### Perceived challenges and constraints to increasing mental health financing

All informants referred to the under-investment in mental health care in Ethiopia, even though there had been important national-level initiatives to expand access to mental health care. The low priority given to mental health was attributed to low awareness and demand for mental health care in the population, low commitment from funders and low buy-in from the Regional Health Bureaus. Difficulties in spending allocated money were also observed, arising from limited capacity to plan and deliver mental health care, resulting in weak programme implementation.

“Non-communicable diseases in general get a very small amount of money, because what is available at a Federal level is not supported by donors, so it, it comes out of the basket fund, the federal ministry monies. So mental health gets a small amount of money, which I believe is not enough. But unfortunately, it has not also been fully utilised, so the small amount of money that has been allocated, has not been fully utilised over set period of the year. So that makes it difficult to ask for more money. …partly the reason is lack of awareness.”

IV01

“Yeah and even one of the tasks that we do is we map resources every year, so one of the areas underfunded is mental health since there are no donors that commit for this area. Maybe after having that data, we will get if there are some of the donors that will be part of that.”

IV11

Respondents were unanimous in identifying the current level of advocacy for mental health as being inadequate, particularly in terms of the clarity and power of the message, the target of advocacy efforts and due to the tendency for advocacy efforts to be one-off rather than persistent. The need to build a better case for mental health care and to engage more effectively with policy-makers was mentioned.

“In addition to working hard, we also need to work smart to ensure the rightful place of mental health in the Ministry of Health bureaucracy. For example, if we take the issues of HIV, there are multiple donors because of special funding sources and visibly people are dying from it. However, unfortunately, we have not been able to articulate how mental illness can be devastating, both to the individual, the family and community and that is has been proven by WHO that the burden is twice as much, 6% versus 13%.”

IV08

As a result of the high level of economic growth in Ethiopia, concern was expressed among participants that this would lead to a decline in external donor funding for mental health initiatives, with some uncertainty about how well mental health programmes would fare when competing for domestic spending. There was also a word of caution about how equitably the growing economy would benefit the broader population, for example, in terms of supporting people’s capacity to pay for insurance and withstand diminishing donor contributions.

“So if we have what we call as proper economic development there is no question that there would be high allocation of resources to health. Because health is one of the pillars, for the, what I mean the multidimensional, index of economic development that… It could because if the economic growth is fairly distributed. You know I mean … the economy is growing. But who is getting what?”

IV12

#### Options for change for increased financing for mental health

Scale-up of community-based health insurance was mentioned by all respondents as being the most important mechanism to increase health financing in general, and mental health care financing in particular. It was stated that CBHI could stimulate increased uptake of health care and accountability of health service delivery, which would in turn necessitate increased governmental financing of health.

“If CBHI is scaled up to a degree where it covers the majority of Ethiopia and informal sector, the plan in the HSTP is to cover 80% of those in the informal sector, so with community-based health care insurance scheme. That will be a game changer for psychiatry. Because you know all drugs are included… so that’s a big opportunity that would be viable and the benefit of CBHI is beyond the financial protection it provides. It brings community empowerment into the system so, when families take their mentally ill patient into the hospital and the drug is not available they will make noises, they will ask questions.”

IV03

Integration of mental health into the health extension programme, a flagship governmental endeavour, as well as the commitment to task-sharing mental health care in primary care facilities, with concomitant expansion of middle cadre mental health specialists, were all expected to increase demand for services, which would in turn drive increased financing.

“…and what makes I think Ethiopia’s case very easy to integrate mental health into the existing system, is the platform is there. The health extension program is there. And the decision to include mental health package as a level 4, you know, health extension program is already there, and 10,000 health extension workers have already been upgraded to level 4. 5,000 more are under training to be level 4. So in 5 years all 38,000 health extension workers will have some level of knowledge and skill about mental health services, that means mental health services become accessible at the community level.”

IV03

A ‘sin tax’ on alcohol and *khat* was mentioned as a possible means to increase financing of health, although current governmental proposals did not specifically earmark the revenue generated by this tax for health.

#### Key elements/criteria for improved public health financing

The need for community engagement was emphasised, in particular to reduce stigma and stimulate demand for mental health care.

“I don’t think we should be simply obsessed in terms of scaling-up services and so forth. Because unless we create the demand, unless we mobilise the community, it doesn’t matter how much, you know we scale-up. It really doesn’t matter because services are not going to be utilised and in the process we are going to lose our credibility. That really worries me and I see that happening now. So to me it’s not being really obsessed about let’s scale-up, let’s scale-up, scale-up. You know if you open up a shop and people are not coming to you to utilise it, it’s going, the Regional Health Bureaus and others are going to say why are we doing this?”

IV08

Some informants also reflected on the need for more mental health-specific health management information system indicators in order to monitor the scale-up of mental health care and demonstrate the need for additional resource allocation. Working through the existing government task-sharing programmes for mental health, in particular the flagship health extension programme was strongly emphasised.

“the biggest platform we have is the health extension program. So you know, integrating mental health into the health extension program. Making sure that health extension workers during their house visits, work with families to support them in terms of their needs, mental health needs. If there is a mentally ill patient to identify that patient early, refer them to the nearest health centre. Make sure that they get treatment there, and as paid workers in the community they have to do the continuous follow-up and so on, to make sure that adherence is there, follow up is there and so on. I mean Ethiopia has already started the journey. My hope is when we look back, we say that what has been designed has actually been implemented.”

IV03

### Sustainable mental health financing: proposed strategies for consideration

Based on the situation analysis, stakeholder reflections and discussions within the Ethiopia team and across the Emerald consortium, the following options for revenue generation, pooling of funds and purchasing of mental health care were identified. These led to a final set of recommended strategic actions for Ethiopia.

#### Generation of funds: increasing revenue sources for mental health

##### Bilateral and multilateral financing

Through governance channels, Ethiopia is doing much of what it can to attract donor funding. It has incorporated mental health into its strategic priorities and health sector plans, signalling to donors that mental health is a priority for Ethiopia. Furthermore, it has defined concrete targets for achieving these priorities, indicating that donor funding for mental health would be strategically spent. Given that such a large proportion (15%) of total health expenditure in Ethiopia comes from external sources, mental health service scale-up would be a candidate for increased donor funding. However, a recent study highlights the bleak reality of donor funding as an option for raising revenue for mental health [66]. Nonetheless, recent World Bank support for investment in mental health as a global development priority is expected to kick-start increased donor commitment to mental health [67].

##### Domestic financing

Calls for increasing domestic funding of mental health are more likely to be met if the overall health budget is increasing, in turn strongly linked to increased government revenue. Options for raising government revenue in Ethiopia include improving tax compliance and efficiency in collection, maximizing revenue from the extraction of a largely untapped natural resource pool, and innovative domestic financing such as diaspora bonds. Recently, the Third International Conference on Financing for Development highlighted the importance of tax revenue generation for financing development [68]. Indeed, Ethiopia’s tax mobilization at 8.8% of GDP is relatively low. Therefore, improving compliance and efficiency in tax collection represents a mechanism for raising government revenue, and ultimately raising revenue for mental health.

##### Innovative financing

Innovative finance for global health is expected to reach US$ 18 billion per year in 2020, and therefore there is an opportunity to tap into these mechanisms as additional support for mental health [21]. Ethiopia may consider options such as mobile health technologies or diaspora bonds.

#### Pooling of funds: enhancing financial access

Out-of-pocket payments, which constitute nine of every ten private dollars spent on health in Ethiopia, are barriers to accessing health services and cause financial hardship. There is consensus that out-of-pocket fees are the least efficient and equitable health financing option, and that funds should be pooled in order to equitably redistribute resources [69]. Ethiopia has opted for a pooling strategy that mixes compulsory health insurance of the formal sector (SHI), and voluntary insurance for the poor and informal sector (CBHI). Given that 85% of the population live in rural areas [70] and pilot studies suggest CBHI to be popular and effective, Ethiopia should continue to prioritise scale up of both CBHI and SHI schemes in health sector plans. However, no country has come close to achieving universal health coverage by using voluntary insurance as its primary financing mechanism [71]. In order to improve financial protection and equity in access, the long-term plan should be to shift people from voluntary schemes into compulsory schemes. This can be done by increasing the number of people in formal employment (thus contributing to SHI) and/or using a tax-based insurance scheme to cover the poor and informal sector.

#### Purchasing: improving efficiency and equity in use of resources

One of the great strengths of the Ethiopian health care system is its extensive coverage of non-specialist health care workers. The national mental health strategy focus on task-sharing mental health care with primary care workers is efficient and pro-poor. However, this initiative needs to be re-invigorated after stalling following expiry of the Strategy and in the wake of low uptake of task-shared care due to inadequate commitment to implementation and lack of community mobilisation [41, 42]. Emerging evidence of successful district level demonstration projects of task-shared care can usefully inform governmental strategy [72, 73]. The roll out of the Ethiopia Primary Health Care Guideline (PHCG) also affords an opportunity for horizontal integration of mental health care, as long as adequate attention is given to the supervision and mentoring of primary care workers by mental health specialists. The addition of a package including mental health care into the upgrading training of the flagship health extension programme demonstrates high level political commitment to expanding access to care, but there needs to be better follow-through of the actual implementation of community-based mental health prevention and promotion activities by health extension workers.

### Recommended strategies

On the basis of all of these available data, information and analysis, three main strategies are proposed for improving public sector performance as well as more equitable financing with respect to mental health care in Ethiopia: (1) increasing efficiency of use of existing resources, (2) increasing revenue for domestic sources and (3) increasing external financing. For each strategy, a number of concrete actions are specified, together with some of the expected advantages and disadvantages associated with them (Table 4).

**Table 4:** Recommended strategies for adequate, fair and sustainable financing of mental health care in Ethiopia

	Strategy elements	Pros and cons
Strategy 1: improve efficiency (use of existing resources)	Ensure that the revision of the National mental health strategy to integrate mental health into primary care is completed and that implementation is re-invigorated Build capacity in mental health care planning and programme implementation at all system levels Implement the new Ethiopia primary health care clinical guidelines (PHCG), which will ensure full horizontal integration of mental health care Mobilise mental health stakeholders to advocate in a consistent and sustained manner for demand generation	*Pros* Ethiopia Primary Health Care Guidelines are likely to have better uptake by regional health bureaus than vertical mental health programmes Community-based mental health care is a wise investment because it reaches more people for less money than hospital-based care Materials for building capacity in mental health care planning have been developed for Ethiopia via the Emerald programme Leveraging existing cadres of health workers to deliver mental health care allows rapid and efficient expansion of human resources for mental health Increasing demand helps to ensure that committed resources are utilised fully and wastage is minimised *Cons* Expanding mental health care, even when integrated into primary care, requires additional money for specialist expansion to provide supervision and training, as well as programme costs Ethiopia PHCG will need to be supported by in-service mental health-specific training because of low level of existing health worker competence Improving efficient use of existing resources does not address the longstanding neglect of mental health care and need for increased resources
Strategy 2: increasing domestic financing for mental health	Inclusion of clearly specified mental, neurological and substance use disorders in the community-based health insurance (CBHI)/social health insurance (SHI) schemes can help to secure increased and more sustainable financing for mental health Introduce a ‘sin tax’ on alcohol and khat and make the case that a proportion of the revenue should be reserved for health in general, and for substance use disorders and mental health specifically Increase revenues by improving tax compliance and efficiency to solidify financing of CBHI/SHI in the longer-term	*Pros* CBHI is equitable and popular at the community level Overcomes key financial barriers to healthcare for people with chronic conditions *Cons* Social health insurance implementation has been delayed The cost to the government of supporting CBHI may be unsustainable and threatened in the event of economic downturn Poorly defined mental disorders within CBHI may undermine the long-term inclusion within insurance packages The increasing burden of chronic disorders may result in unaffordable tariffs for the community and governmental underpinning of CBHI and SHI Lobbying from special interest groups may lead to unfair distribution of domestic finances Money from the ‘sin tax’ may not be equitably distributed
Strategy 3: increase external financing	Demonstrate how mental health is relevant for the global agenda on NCDs, chronic communicable diseases (HIV and TB) and maternal health to strengthen the investment case for mental health care Demonstrate how mental health is relevant for SDG target for UHC to leverage external funding for mental health care External support for scale-up of Ethiopia PHCG will lead to increased external support for mental health care scale-up Advocate for inclusion of financing for mental health care in core international development aid for low-income countries, building on World Bank commitments to mental health Advocate for establishment of a global fund for mental health to focus on people with severe mental disorder	*Pros* Focus on co-morbidity promotes a joining up of thinking of physical and mental health Ethiopia-specific evidence exists for the relevance of mental health to public health and development priorities Targeted advocacy can leverage big funds from changing global priorities International funders can kick-start an agenda for inclusion of people with mental health problems in low-income countries A global fund can address the chronic neglect of care for persons with severe mental disorder *Cons* Focus on depression co-morbidity runs the risk that severe mental illness is neglected because of low prevalence (1–2%) even though high disability and human rights burden External funding of health in Ethiopia will decrease concomitantly with the transition to middle-income country status

## Discussion

In this paper we have integrated findings from a conceptually-driven comprehensive situation analysis and in-depth interviews with key stakeholders to make recommendations for moving towards universal health coverage for mental disorders in Ethiopia. The recommendations focus on strategies for adequate, fair and sustainable financing of mental health care coupled with expanded service coverage.

The Ethiopian government has demonstrated its commitment to the expansion of mental health care but efforts to date have been hampered by low financing (combined with low budget absorption due to challenges with implementation and scale-up of mental health care) and inadequate financial risk protection of people seeking care [15]. The most hopeful strategy we identified to achieve UHC for people with mental disorders is through Ethiopia’s commitment to pooled risk protection through public health insurance. Expansion of public health insurance has the potential to drive increased service coverage and quality of care, by specifying a minimum package of evidence-based care that can be expected at a particular level in the health system, as well as protecting people from the impoverishing effects of high out-of-pocket health expenditure.

Leveraging of existing resources by showing how mental health is relevant to broader public health and development concerns is often touted as a means by which mental health care can be expanded [74]. In Ethiopia, there is ample evidence of the broader relevance of mental health to maternal and child health outcomes and uptake of reproductive health care [75–77], the course and outcomes of people with chronic illnesses such as tuberculosis [78], HIV [79], non-communicable diseases [80], poverty reduction strategies [11] and child educational outcomes [81]. The new Ethiopia primary health care guidelines, which are a contextualised version of the South African Practical Approach to Care Kit [82], have enabled mental health to be effectively integrated into the clinical guidelines for most of the common presenting complaints in primary care. This horizontal integration of mental health care does indeed hold promise for expanding care for the common mental disorders, particularly depression and anxiety, and substance use disorders; however, this approach does little to expand financing of care for people with severe mental disorders, such as schizophrenia and bipolar disorder [83]. Although severe mental disorders are associated with high levels of individual burden and human rights abuses, the prevalence is low and demand for care is complicated by the nature of the condition which can interfere with help-seeking behaviours [15]. In view of these particular challenges in providing health care for people with severe mental disorders, there have been calls for establishment of a global fund [84].

Our key informants emphasised the need for effective advocacy to secure increased financing for mental health care in Ethiopia, pointing at the success of people living with HIV. Historically, the voices of people with mental health problems in Ethiopia have had little opportunity to be heard. Until recently there was just one national organisation of caregivers of people with mental health problems and little grass roots representation. A baseline qualitative study in a population where primary mental health care was being implemented indicated that there were low levels of awareness about mental health treatments and the quality of care that people could rightfully expect, low levels of mobilisation and empowerment to engage in advocacy and structural barriers of stigma and poverty that precluded involvement in improving mental health services [85]. However, there was appetite from people with mental health problems and their caregivers to contribute to improving and expanding mental health care. As part of Emerald, capacity-building materials have been developed and employed to equip both service users and healthcare providers to strengthen service user involvement in mental health system strengthening, and in advocating for adequate resources for mental health [86].

Efforts to realise the scale-up of access to mental health care and improve the mental health of populations in LMICs cannot achieve success without attention to the necessary health system supports [16]. However, there is very little evidence to support governments to develop strategies to finance scaled up mental health care. The Emerald framework employed in this paper can be applied across other settings in sub-Saharan Africa, with the likelihood that common challenges and strategies will emerge and can be used to strengthen the collective voice at the regional level.

Strengths of our study include the theoretical approach to data collation and synthesis, triangulation of data from multiple services, including interviews with some of the key players in (mental) health care scale-up and financing in Ethiopia, contextualisation of recommendations to the constraints and opportunities of the Ethiopian context and our focus on practical actions. A limitation was the small number of key informants that we were able to access and the lack of independent coding of the qualitative data.

## Conclusion

In this paper we have developed contextualised recommendations for Ethiopia to achieve universal health coverage for mental health care. All the three proposed strategies are needed for success. Easy wins may be the leveraging of resources from high-priority SDG-related programmes and implementation of existing plans to support task-shared mental health care. However, these actions need to be supported by the continued expansion of public health insurance to ensure that uptake of mental health care is equitable and progressive. Given the low base from which mental health care coverage is starting, external donors also need to play a role and deliver on SDG commitments to include mental health within development funding. Future researchers and planners can apply the approach described in this paper to other countries of sub-Saharan Africa and identify common strategies for sustainable and equitable financing of mental health care.

## Additional files

**Additional file 1.** Situation analysis template.

**Additional file 2.** Literature search terms.

**Additional file 3.** PRISMA flow diagram.

## Abbreviations

CBHI: community-based health insurance
DALY: disability adjusted life years
EMERALD: emerging mental health systems in low- and middle-income countries
FMOH: Federal Ministry of Health
GDP: gross domestic product
GGE: general government expenditure
GGHE: general government health expenditure
HSDP: health sector development plan
HSTP: health sector transformation plan
NCD: non-communicable disease
NGO: non-governmental organization
NHP: national health policy
NMHS: national mental health strategy
PHC: primary health care
SDG: sustainable development goal
SHI: social health insurance
THE: total health expenditure
YLD: years lived with disability
