# Supplementary material for: Moving towards universal health coverage for mental disorders in Ethiopia
Source: Int J Ment Health Syst. 2019 Feb 25;13:11. doi: 10.1186/s13033-019-0268-9 (PMC6388484; doi:10.1186/s13033-019-0268-9)
Supplement: Supplementary file 2 — Additional file 2. Literature search terms. [file 13033_2019_268_MOESM2_ESM.docx]

Additional file 2: Search Terms, Search Strategy and Results of literature review

1. UHC related terms

Universal health coverage, UHC, Universal health care, healthcare financing, health care expenditure, out of pocket spending, out of pocket expenditure, catastrophic health expenditure, catastrophic health spending

"healthcare financing"[MeSH Terms]

"Insurance, Health"[Mesh]

"National Health Programs"[Mesh]

2. Country

Ethiopia

"Ethiopia"[Mesh]

21 June 2018

*PubMed Search*

#1

universal health care[tiab] OR universal health coverage[tiab] OR UHC[tiab] OR "healthcare financing"[mesh] OR healthcare finance*[tiab] OR health care finance*[tiab] OR health finance*[tiab] OR "Insurance, Health"[mesh] OR "National Health Programs"[mesh] OR health insurance[tiab] OR national health program*[tiab] OR community based health insurance[tiab] OR social health insurance[tiab] OR health care expenditure*[tiab] OR out?of?pocket spending*[tiab] OR out?of?pocket expenditure*[tiab] OR catastrophic health expenditure*[tiab] OR catastrophic health spending*[tiab] OR universal health care[tw] OR universal health coverage[tw] OR UHC[tw] OR "healthcare financing"[mesh] OR healthcare finance*[tw] OR health care finance*[tw] OR health finance*[tw] OR "Insurance, Health"[mesh] OR "National Health Programs"[mesh] OR health insurance[tw] OR national health program*[tw] OR community based health insurance[tw] OR social health insurance[tw] OR health care expenditure*[tw] OR out?of?pocket spending*[tw] OR out?of?pocket expenditure*[tw] OR catastrophic health expenditure*[tw] OR catastrophic health spending*[tw]

(Results: 241880)

#2

"Ethiopia"[mesh] OR Ethiopia*[tiab] OR Ethiopia*[tw]

(Results: 14041)

#3

#1 AND #2

(Results: 96)

*Medline Search (via EBSCO)*

S1

universal health care OR universal health coverage OR uhc OR healthcare financing OR health financing OR health insurance OR community based health insurance OR social health insurance OR health care expenditure OR out of pocket spending OR out of pocket expenditure OR catastrophic health expenditure OR catastrophic health spending

(Results: 88282)

S2

ethiopia

(Results: 15246)

S3

S1 AND S2

(Results: 95)

Out of the 96 PubMed search results, one is duplicate leaving the final unique search results 95. Similarly, the final Medline search results were 94 after removing one duplicate. Out of the remaining 94 results, 54 were overlapping with PubMed results while the other 40 results were unique results from Medline. Taken together, there were 135 search results to screen.
