# Supplementary material for: Moving towards universal health coverage for mental disorders in Ethiopia
Source: Int J Ment Health Syst. 2019 Feb 25;13:11. doi: 10.1186/s13033-019-0268-9 (PMC6388484; doi:10.1186/s13033-019-0268-9)
Supplement: Supplementary file 3 — Additional file 3. PRISMA flow diagram. [file 13033_2019_268_MOESM3_ESM.docx]

PRISMA flow diagram of study selection for literature review

Records identified through database searching
n = 191

(*PubMed*=96, *Medline*=95)

## Identification

Full-text articles excluded on the basis of framework dimensions of the study
(n = 11)

Records excluded with title and abstract screen
(n = 120)

Full-text articles assessed for eligibility
(n = 15)

Studies included in the literature review
(n = 4)

## Included

Records screened
(n = 135)

Records after duplicates removed
(n = 56)

## Eligibility

## Screening
